# Synthetic oleanane triterpenoids enhance blood brain barrier integrity and improve survival in experimental cerebral malaria

**DOI:** 10.1186/s12936-017-2109-0

**Published:** 2017-11-14

**Authors:** Valerie M. Crowley, Kodjo Ayi, Ziyue Lu, Karen T. Liby, Michael Sporn, Kevin C. Kain

**Affiliations:** 1 S. A. Rotman Laboratories, Sandra Rotman Centre for Global Health, University Health Network-Toronto General Hospital, Toronto, Canada; 2 Department of Pharmacology, Dartmouth Medical School, Hanover, NH USA; 3 Department of Medicine, University of Toronto, Toronto, ON Canada; 4 Tropical Diseases Unit, Division of Infectious Diseases, Department of Medicine, UHN-Toronto General Hospital, Toronto, ON Canada

## Abstract

**Background:**

Cerebral malaria (CM) is a severe complication of *Plasmodium falciparum* infection associated with high mortality and neurocognitive impairment in survivors. New anti-malarials and host-based adjunctive therapy may improve clinical outcome in CM. Synthetic oleanane triterpenoid (SO) compounds have shown efficacy in the treatment of diseases where inflammation and oxidative stress contribute to pathogenesis.

**Methods:**

A derivative of the SO 2-cyano-3,12-dioxooleana-1,9-dien-28-oic acid (CDDO), CDDO-ethyl amide (CDDO-EA) was investigated for the treatment of severe malaria in a pre-clinical model. CDDO-EA was evaluated in vivo as a monotherapy as well as adjunctive therapy with parenteral artesunate in the *Plasmodium berghei* strain ANKA experimental cerebral malaria (ECM) model.

**Results:**

CDDO-EA alone improved outcome in ECM and, given as adjunctive therapy in combination with artesunate, it significantly improved outcome over artesunate alone (p = 0.009). Improved survival was associated with reduced inflammation, enhanced endothelial stability and blood–brain barrier integrity. Survival was improved even when administered late in the disease course after the onset of neurological symptoms.

**Conclusions:**

These results indicate that SO are a new class of immunomodulatory drugs and support further studies investigating this class of agents as potential adjunctive therapy for severe malaria.

## Background

Malaria remains the most important parasitic disease in the world, causing an estimated 225 million cases and 438,000 deaths annually [1]. The greatest burden of severe and fatal disease is borne by children mainly in sub-Saharan Africa [1, 2]. Current elimination efforts have considerably reduced the global malaria burden, however these advances may paradoxically increase the risk of severe and fatal disease as the level of clinical immunity in the population wanes due to decreased parasite prevalence [3, 4].

The mechanisms underlying cerebral malaria (CM) are incompletely understood, however an interaction between both parasite virulence factors and host response determinants play critical roles in the pathobiology of CM. Parasitized erythrocytes sequester in the microvasculature of vital organs, including the brain, and lead to host innate immune responses, including the release of pro-inflammatory cytokines and angiogenic factors, culminating in a dysregulated inflammatory response, endothelial dysfunction and loss of blood brain barrier (BBB) integrity [5–8]. C57BL/6 mice infected with *Plasmodium berghei* strain ANKA serves as an experimental model of cerebral malaria (ECM), where mice develop severe vasculopathy and neurological manifestations [5, 9–13]. While some features differ between ECM and human CM, those involving microvascular leakage and BBB dysfunction are especially comparable in mice and humans during ECM and human CM, respectively [14].

Parenteral artesunate is the current first-line therapy for CM; however, the fatality rate of CM remains high at 18–30%, with up to one-third of survivors developing long-term neurocognitive impairments [15–19]. Furthermore, the current efficacy of parenteral artesunate is threatened by the emergence and spread of artemisinin resistance in Southeast Asia [20]. New classes of anti-malarial drugs and new strategies for adjunctive therapy are urgently needed to improve survival and decrease the burden of neurological injury in survivors.

Triterpenoids are a structurally diverse class of organic molecules naturally synthesized by many plants by the cyclization of squalene [21]. Some naturally occurring triterpenoids, such as ursolic and oleanolic acid, possess weak antitumour and anti-inflammatory properties [22, 23]. To improve their potency as pharmacological agents, synthetic oleanane triterpenoids (SO) have been synthesized and represent a promising class of multifunctional drugs that modulate the activity of several signalling networks [24].

SO are potent activators of host immunomodulatory pathways, including nuclear factor-erythroid2-related factor 2 (Nrf2) both in vitro and in vivo [25]. Under homeostatic conditions Nrf2 is bound by its endogenous inhibitor, Kelch-like ECH-associated protein 1 (Keap1) in the cytoplasm, an interaction that targets the complex for ubiquitination and proteasomal degradation. Under conditions of oxidative stress the Nrf2-Keap1 complex dissociates, allowing Nrf2 to translocate into the nucleus, bind to antioxidant response element (ARE) sequences and induce the transcription of anti-oxidant and anti-inflammatory factors that function to reduce toxicity. SO activate the Nrf2/ARE pathway by directly binding to Keap1, thereby activating Nrf2 [26]. Genes containing ARE sequences include haem oxygenase (HO-1), catalase, superoxide dismutase, and nuclear transcription factors, such as peroxisome proliferator-activating receptor (PPARγ), highlighting the pleiotropic actions of SO [27–29].

Based on in vitro observations and their ability to target numerous pathways, SO have the potential to be broadly applied to chronic and infectious diseases where host inflammation and oxidative stress contribute to pathogenesis. The methyl ester of a potent SO, 2-cyano-3,12-dioxooleana-1,9 (11)-dien-28-oic acid (CDDO-Me) and related triterpenoids are currently in clinical trials for pulmonary arterial hypertension (clinicaltrials.gov NCT02036970, NCT02657356), Alport Syndrome (NCT03019185) and chronic kidney disease (NCT01053936), and various forms of cancer [30, 31]. Other derivatives, including CDDO-ethyl amide (CDDO-EA) have been formulated for improved bioavailability and to better cross the BBB [32, 33]. While the pathogenesis of malaria is complex, it is characterized by marked inflammation, oxidative stress and their impact on microvascular function [34].

It was hypothesized that SO would improve outcome alone or as combination therapy in the treatment of severe malaria. To test this hypothesis, the effect of CDDO-EA was examined in vivo in a murine model of ECM. This study tested for the effect of CDDO-EA on host response including inflammation, endothelial dysfunction and BBB leak.

## Methods

### Mice

The University Health Network Animal Use Committee approved all experiments in accordance with institutional guidelines (AUP 1920). Male and female 7–9 weeks old C57BL/6 mice were purchased from Jackson Laboratories (West Grove, PA, USA) and housed under pathogen-free conditions with a 12-h light cycle.

### Infection with *Plasmodium berghei*

*Plasmodium berghei* ANKA (PbA) parasites (MR4; American Type Culture Collection, Manassas, VA, USA) were cultivated by passage through C57BL/6J mice as described [35]. Experimental infections were initiated by intraperitoneal (ip) injection of 1 × 10^6^ parasitized erythrocytes (day 0). Parasite burdens were monitored daily starting on day 3 post-infection for up to 14 days by Giemsa-stained blood smears. Mice were inoculated on the same day, within 30 min and inoculation alternated between control and experimental groups (e.g. control 1, experimental 1, control 2, experimental 2). Mice were assessed for signs of ECM using a modified rapid murine coma and behaviour scale (RMCBS) [36] and were euthanized when moribund according to institutional guidelines [37].

### Reagents and drug treatments

For in vivo experiments, CDDO-EA [25, 32] was dissolved in vehicle containing DMSO, Cremophor EL (Sigma-Aldrich) and PBS at a 1:1:8 ratio. Adjunctive therapy with CDDO-EA preparation (200 μmol/kg) and artesunate (5 mg/kg; Sigma-Aldrich) were co-administered. All treatments were a single dose, administered by an ip injection, in a total volume of 0.2 mL. Treatment was initiated on day 4 or 6 post-infection. Treatment on day 4 corresponds to established infection prior to the development of severe disease, whereas treatment on day 6 corresponds to the onset of neurological manifestations (after BBB leak and the onset of neurological symptoms) [37] defined as a RMBCS of ≤ 50%. On day 6, clinical manifestations of ECM include ruffled hair, hunched body position, inactivity, poor balance, reduced limb strength, reduced touch escape and ataxic gait [36].

### Analysis of blood brain barrier integrity

On day 6 post-infection, mice were injected ip with 300 µL of 2% Evans blue. After 2 h, mice were euthanized using isofluorane, and perfused with 20 ml of PBS. Brains were collected, photographed and placed in formamide for 48 h to extract Evans blue dye. Evans blue dye was quantified using a spectrophotometer at 605 nm and compared to a standard curve.

### Measurement of transcript levels

Transcripts were measured by quantitative real-time PCR (qRT-PCR). Total RNA was isolated from snap frozen brain tissue after homogenization in TRIzol (1 mL/100 mg tissue; Invitrogen, Burlington, ON, USA) according to manufacturer’s instructions. Extracted RNA (1 μg/sample) was treated with DNase I (Fermentas, Burlington, ON, USA), and reverse transcribed to cDNA (BioRad, Mississauga, ON, USA). cDNA was amplified in triplicate with SYBR Green master mix (Roche, Laval, QC, Canada) in the presence of 1 μM of forward and reverse primers in a Light Cycler 480 (Roche, Laval, QC, Canada). Transcript expression levels were calculated compared to a standard curve of mouse genomic DNA included on each plate and normalized to average GAPDH expression levels. The primer used are as follows: for Nrf-2 (mouse), 5′-TCTCCTCGCTGGAAAAAGAA-3′ and 5′AATGTGCTGGCTGTGCTTTA-3′ and for HO-1 (mouse), 5′-AACAAGCAGAACCCAGTCTATGC-3′ and 5′-AGGTAGCGGGTATATGCGTGGGCC-3′, GAPDH (mouse) 5′-TCAACAGCAACTCCCACTCTTCCA-3′ and 5′-TTGTCATTGAGAGCAATGCCAGCC-3′, GAPDH (human) 5′-GCCTCAAGATCATCAGCAATGC-3′ and 5′-CCTTCCACGATACCAAAGTTGTCAT-3′, Nrf-2 (human) 5′-CCTCAACTATAGCGATGCTGAATCT-3′ and 5′-AGGAGTTGGGCATGAGTGAGTAG-3′, HO-1 (human) 5′-GCAGAGAATGCTGAGTTCATG-3′ and 5′-CACATCTATGTGGCCCTGGAGGAGG-3′.

### Measurement of cytokines and markers of endothelial activation

Cytokines and markers of endothelial activation were measured using an enzyme-linked immunosorbent assay (ELISA) on day 6 post-infection on mice that were drug-treated on day 4 post-infection. Blood was collected in heparin-coated tubes by cardiac puncture and assayed for interleukin-10 (IL-10), tumour necrosis factor (TNF), interferon-γ (IFN-γ), and angiopoietin-1 (Ang-1) using commercially available standard ELISA kits according to the manufacturer’s instructions (Duosets, R&D Systems, Minneapolis, MN, USA). Ang-2 was measured using a Quantikine ELISA kit (R&D Systems, Minneapolis, MN, USA).

### Isolation of peripheral blood mononuclear cells

Human peripheral blood mononuclear cells (PMBCs), 5 × 10^5^ cells/well were treated with 1 μM dihydro-artemisinin (DHA) dissolved in DMSO. After incubation for 24 h at 37 °C, supernatants were removed and cells were resuspended directly in TRIzol for Nrf2 and HO-1 mRNA quantification by qRT-PCR. Fresh PBMCs were obtained from healthy blood donors. All blood donors were provided with written informed consent by a protocol approved by the University of Toronto, Protocol Reference #21081.

### Statistical analysis

A log-rank test was performed on the Kaplan–Meier survival curves. The Kruskal–Wallis H test was performed on RMBCS, mRNA levels, ELISAs and Evans blue data and multiple comparisons were corrected using Dunn’s correction. A *t* test was carried out on the transcript levels from PBMCs. Tests were performed with Prism version 7.0b (Macintosh version GraphPad Software Inc, La Jolla, CA, USA).

## Results

### Mono- and adjunctive CDDO-EA therapy improves outcome in experimental cerebral malaria

**Fig. 1:**
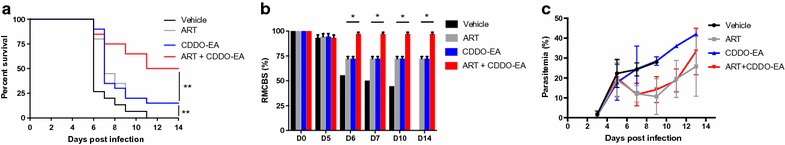
Single dose CDDO-EA improves survival and prevents the development ECM without affecting parasitaemia. Seven to 9 weeks-old C57BL/6J mice were treated once, on day 4 post-infection, by intraperitoneal (ip) injection. CDDO-EA was administered at 200 μmol/kg, ART at 5 mg/kg. **a** Therapeutic treatment with CDDO-EA monotherapy significantly improved survival compared to vehicle control alone (χ^2^ = 7.70, p = 0.006, by log-rank test). Adjunctive therapy of CDDO-EA significantly improved survival above artesunate therapy alone (χ^2^ = 6.75, p = 0.009, by log-rank test, n = 15-20 mice per group). Data combined from two independent studies. **b** Treatment with CDDO-EA significantly reduced disease severity as determined by the RMBCS compared to treatment with artesunate alone 6, 7, 10, and 14 days post-infection (p = 0.048, by Kruskal–Wallis with Dunn’s correction, n = 5 mice per group). **c** Treatment with CDDO-EA had no impact on parasite burden. Parasitaemia was monitored using thin blood smears stained with Giemsa. CDDO-EA did not reduce parasitaemia

To determine whether therapeutic administration of CDDO-EA would improve outcome in C57BL/6J mice susceptible to ECM, PbA-infected mice were either treated with a single dose of CDDO-EA alone or in combination with artesunate. CDDO-EA monotherapy on day 4 post-infection significantly improved survival compared to vehicle control (p = 0.006; Fig. 1a). Survival with CDDO-EA monotherapy was equivalent to that of mice treated with artesunate alone. When single dose CDDO-EA was administered adjunctively with artesunate on day 4 post-infection, survival was significantly improved over that of artesunate treatment alone (p = 0.009; Fig. 1a). Mice treated with CDDO-EA in combination with artesunate did not develop neurological manifestations of ECM as assessed by RMCBS (Fig. 1a). The improved survival and reduced disease severity with CDDO-EA intervention was not associated with changes in parasite burden as determined by peripheral parasitaemia (Fig. 1c).

### CDDO-EA monotherapy increases *Nrf2* and *HO*-*1* levels but is counteracted by artemisinin

SO are potent activators of the transcriptional regulator Nrf2 [25] and HO-1, a transcriptional target of Nrf2, has been implicated in the pathogenesis of ECM [38]. Therefore, the levels of *Nrf2* and *HO*-*1* transcripts were assessed in the brains of PbA-infected mice on day 6 post-infection.

**Fig. 2:**
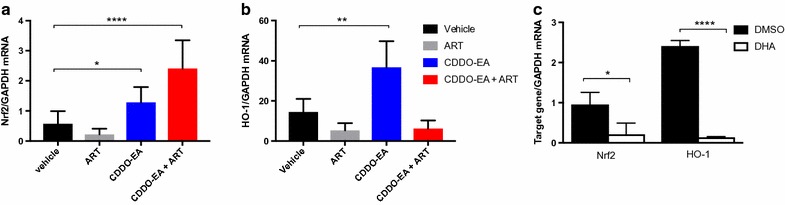
CDDO-EA increases the transcription of *Nrf2* and *HO*-*1* in the brains of mice infected with malaria parasites, whereas artemisinin inhibit *Nrf2* and *HO*-*1* expression in murine brains and in human PBMCs. CDDO-EA was administered at 200 μmol/kg, and ART at 5 mg/kg on day 4 post-infection, with 17–22 mice per group, data combined from two independent studies. **a** CDDO-EA mono (p = 0.030) and adjunctive treatment (p < 0.0001) significantly increases *Nrf2* expression in the brains of malaria-infected mice (Kruskal–Wallis test with Dunn’s correction, respectively). A trend for decreasing *Nrf2* expression with artesunate monotherapy was observed. **b** CDDO-EA monotherapy significantly increases levels of *HO*-*1* transcripts (p = 0.007 Kruskal–Wallis test with Dunn’s correction). Artesunate monotherapy results in significantly reduced expression of *HO*-*1* (p = 0.002, Kruskal–Wallis test with Dunn’s correction). **c** DHA (1 μM), the active component of all artemisinins significantly reduces the expression of both *Nrf2* and *HO*-*1* in human PBMCs (p = 0.035, < 0.0001, *t* test, respectively). Experiments were performed in triplicate

Increased levels of *Nrf2* and *HO*-*1* transcripts were observed in mice treated with CDDO-EA monotherapy (p = 0.030) and CDDO-EA in combination with artesunate (p ≤ 0.0001), whereas artesunate alone decreased the levels of *Nfr2* and *HO*-*1* compared to vehicle alone (Fig. 2a, b).

To determine whether the inhibitory effects of artesunate on *Nrf2* and *HO*-*1* expression were also observed in human cells, PBMCs were incubated with and without DHA, the active metabolite of artemisinins. PBMCs incubated with DHA had significantly reduced expression of *Nrf2* and *HO*-*1* compared to control-treated cells (p = 0.035, < 0.0001, respectively, Fig. 2c).

### Mono- and adjunctive CDOO-EA therapy reduces cytokine levels and improves endothelial stability

**Fig. 3:**
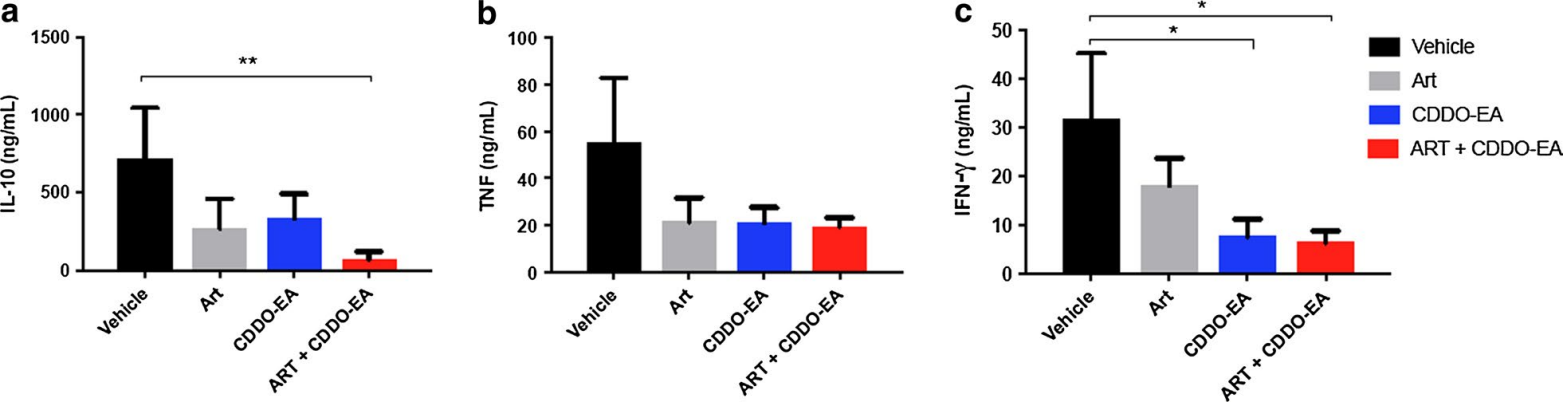
Effect of CDDO-EA mono- and adjunctive therapy on cytokines during ECM. Factors were measured in plasma by ELISA on day 6 post-infection. Treatments were administered on day 4 post-infection (CDDO-EA administered at 200 μmol/kg, ART at 5 mg/kg, 4–5 mice per group, data from two independent studies). **a** Significantly lower levels of IL-10 were observed with adjunctive CDDO-EA therapy compared to vehicle alone (p = 0.003, Kruskal–Wallis test with Dunn’s correction). **b** A trend towards lower plasma TNF was observed on TNF levels with CDDO-EA therapy. **c** Significantly lower levels of IFN-γ levels were observed between CDDO-EA monotherapy (p = 0.032, Kruskal–Wallis test with Dunn’s correction) and adjunctive CDDO-therapy (p = 0.018, Kruskal–Wallis test with Dunn’s correction) compared to vehicle alone

**Fig. 4:**
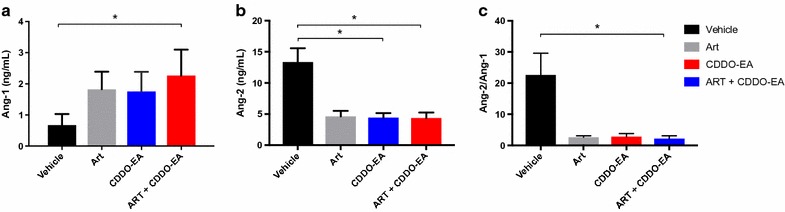
CDDO-EA treatment reduced malaria-induced endothelial activation as determined by levels of angiopoeitins in plasma. Factors were measured by ELISA on day 6 post-infection. Treatments were administered on day 4 post-infection (CDDO-EA administered at 200 μmol/kg, ART at 5 mg/kg, 4–5 mice per group, data from two independent studies). **a** Co-administration of CDDO-EA and artesunate significantly increased levels of plasma Ang-1 (p = 0.014, Kruskal–Wallis test with Dunn’s correction). **b** Monotherapy and co-administration of CDDO-EA with artesunate significantly decreased levels of plasma Ang-2 (p = 0.030, 0.049, Kruskal–Wallis test with Dunn’s correction, respectively). **c** Co-administration of CDDO-EA with artesunate significantly reduced the ratio of Ang-2/Ang-1 (p = 0.012, Kruskal–Wallis test with Dunn’s correction)

Immune and endothelial dysfunction have been implicated in the pathogenesis of CM in humans and in ECM [8]. Dysregulation of plasma angiopoietin levels is associated with disease severity and predicts clinical outcome in human malaria [37, 39–41]. Increased survival of mice treated adjunctively with CDDO-EA was associated with decreased levels of plasma IL-10 (p = 0.003), TNF and IFN-γ (p = 0.018, Fig. 3). CDDO-EA treatment alone and in combination with artesunate resulted in enhanced endothelial stability [37] as determined by increased Ang-1 (adjunctive, p = 0.014), decreased Ang-2 levels (p = 0.030 and 0.049, monotherapy and adjunctive therapy respectively) and lower Ang-2/Ang-1 ratio compared to vehicle alone (adjunctive, p = 0.012, Fig. 4).

### Mono- and adjunctive CDDO-EA therapy enhances BBB integrity

**Fig. 5:**
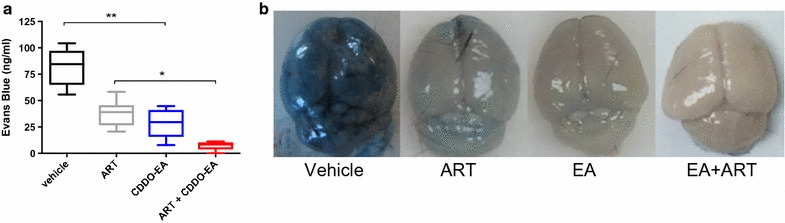
CDDO-EA treatment enhances BBB integrity. Evans blue dye was measured by spectrophotometer on day 6 post-infection. Treatments were administered on day 4 post-infection (CDDO-EA administered at 200 μmol/kg, ART at 5 mg/kg, 9 mice per group, data from two independent studies). **a** CDDO-EA monotherapy significantly reduced BBB leak compared to vehicle control alone (p = 0.009, Kruskal–Wallis test with Dunn’s correction) and co-administration of CDDO-EA with artesunate significantly reduced BBB extravasation compared to treatment with artesunate alone (p = 0.018, Kruskal–Wallis test with Dunn’s correction). **b** Representative images of brains of PbA-infected mice and their respective treatments. Images reflective of population medians are shown

BBB leak is a hallmark of both ECM and paediatric CM [14, 42–45]. The improved survival of PbA-infected mice treated with CDDO-EA alone and in combination with artesunate was associated with preservation of the BBB as assessed by quantification of Evans blue extravasation into brain parenchyma (Fig. 5a, b). Compared to untreated mice, CDDO-EA alone significantly reduced cerebrovascular leak compared to vehicle control (p = 0.009). This reduction in leak was comparable to that obtained with artesunate treatment. Adjunctive CDDO-EA enhanced BBB integrity beyond that obtained with artesunate treatment alone (p = 0.018).

### Adjunctive CDDO-EA treatment improves survival compared to artesunate alone when administered to mice with neurological impairment

**Fig. 6:**
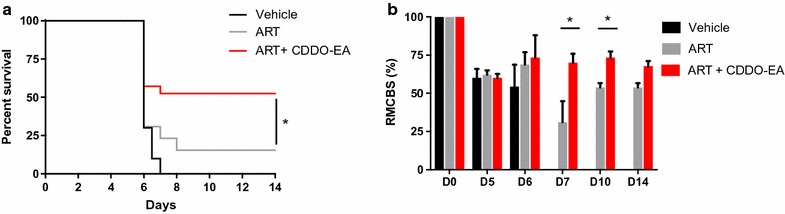
Adjunctive CDDO-treatment improves survival and neurological outcome over anti-malarial therapy alone. Seven to 9 weeks-old C57BL/6 mice were treated, on day 6 post-infection by single dose ip injection, after the onset of neurological symptoms of ECM. Data combined from two independent studies. **a** A significant difference in survival was observed between artesunate monotherapy and adjunctive CDDO-EA treatment (χ^2^ = 4.49, p = 0.0341, by log-rank test, n = 15–20 mice per group). **b** Significantly better neurological outcomes as determined by the RMBCS were observed for adjunctive CDDO-treatment compared to artesunate monotherapy (p = 0.032, 0.048, Mann–Whitney U test, 5 mice per group). RMBCS for day 6 were measured 16 h after injection

To better mimic a clinically relevant scenario when children present with neurological impairment, mice were treated with CDDO-EA alone and adjunctively at the onset of neurological symptoms. Survival was significantly improved with adjunctive CDDO-EA treatment compared to artesunate alone (p = 0.0341, Fig. 6a). Mice receiving adjunctive CDDO-EA also displayed significantly reduced disease severity at days 7 and 10 post-infection as determined by the RMCBS ((p = 0.032 and 0.048 respectively, Fig. 6b).

## Discussion

SO are a class of drugs that has shown promise in the treatment of chronic, non-communicable diseases where pathogenesis is linked to inflammation and oxidative stress [25]. A pre-clinical model of severe malaria, shows the potentially utility of CDDO-EA as an adjunctive therapy for a life-threatening infection.

SO regulate Nrf2-activity by releasing its interaction with its inhibitor Keap-1, allowing for the translocation of Nrf2 to the nucleus and for the transcription of Nrf2 target genes [24, 46, 47]. An observed increase in *Nrf2* transcription in the brains of PbA-infected mice upon CDDO-EA treatment, likely reflects autoregulation of Nrf2 that has been observed in vitro and in kidneys of mice treated with CDDO-Im [48, 49]. CDDO-EA was administered only once and this Nrf2 positive feedback loop may be beneficial in prolonging the protective effects of a single dose of CDDO-EA. Of particular interest to ECM is the Nrf2-target gene *HO*-*1*, which has been shown to modify the pathogenesis of ECM and improve outcome and CDDO-EA treatment alone increased levels of *HO*-*1* [38]. The data suggests that adjunctive CDDO-EA therapy improves survival though a Nrf2-dependent, HO-1 independent mechanism. However importantly, artesunate and DHA, first-line therapy for human falciparum malaria, inhibit *Nrf2* and *HO*-*1* expression in both human PBMCs and in malaria-infected mice. Of note, co-treatment with CDDO-EA overcomes the inhibition of *Nrf2* by artemisinin but not that of *HO*-*1* in ECM. Whether the decrease of *Nrf2* and *HO*-*1* expression associated with artemisinin treatment has implications for cellular recovery, detoxification and neurological outcomes in severe and CM in humans remains to be determined but theoretically inhibiting these pathways could have a negative impact on malaria outcomes [38, 49, 51, 70, 71]. Clinical trial evidence indicates that neurocognitive deficits in children treated with parenteral artesunate for CM remain high at up to 35% of survivors [50] and it will be of interest to evaluate whether CDDO-EA treatment can mitigate this high level of neurological impairment in pre-clinical or ultimately clinical studies [51].

The immuno-regulatory cytokine IL-10 plays a protective role in the development of ECM [52–54]. CDDO-EA therapy has been shown to increase levels of IL-10 in an experimental murine model of chronic liver injury [55]. Although CDDO-EA improves the survival of ECM-susceptible mice, it appears to do so through an IL-10-independent mechanism, as treatment reduced IL-10 levels in PbA-infected mice. Although high levels of IL-10 may play a beneficial role in human malaria by inhibiting parasite-induced pro-inflammatory responses that contribute to disease severity [56], studies have [57, 58] reported that high levels may also impair malaria clearance [59]. The development of severe malaria is associated with a dysregulated host immune response to infection characterized by increased circulating levels of TNF and IFN-γ in both humans [60–63] and murine models of ECM [54]. To determine if CDDO-EA was able to modify the host inflammatory response, the levels of these pro-inflammatory cytokines were assessed in ECM. CDDO-EA treatment alone led to significantly reduced levels of both IFN-γ and TNF, in agreement with previous observations in a murine model of cirrhosis [55]. It is possible that a single time point fails to capture crucial dynamic differences of these mediators between experimental groups, and most mice died on day 7 post-infection rather than day 6 when the measurements were made. Of note anti-inflammatory adjunctive therapies (e.g. dexamethasone, anti-TNF strategies) have been shown to improve outcome of human CM; with some reported to result in prolonged coma and worse clinical outcomes [64–68].

**Fig. 7:**
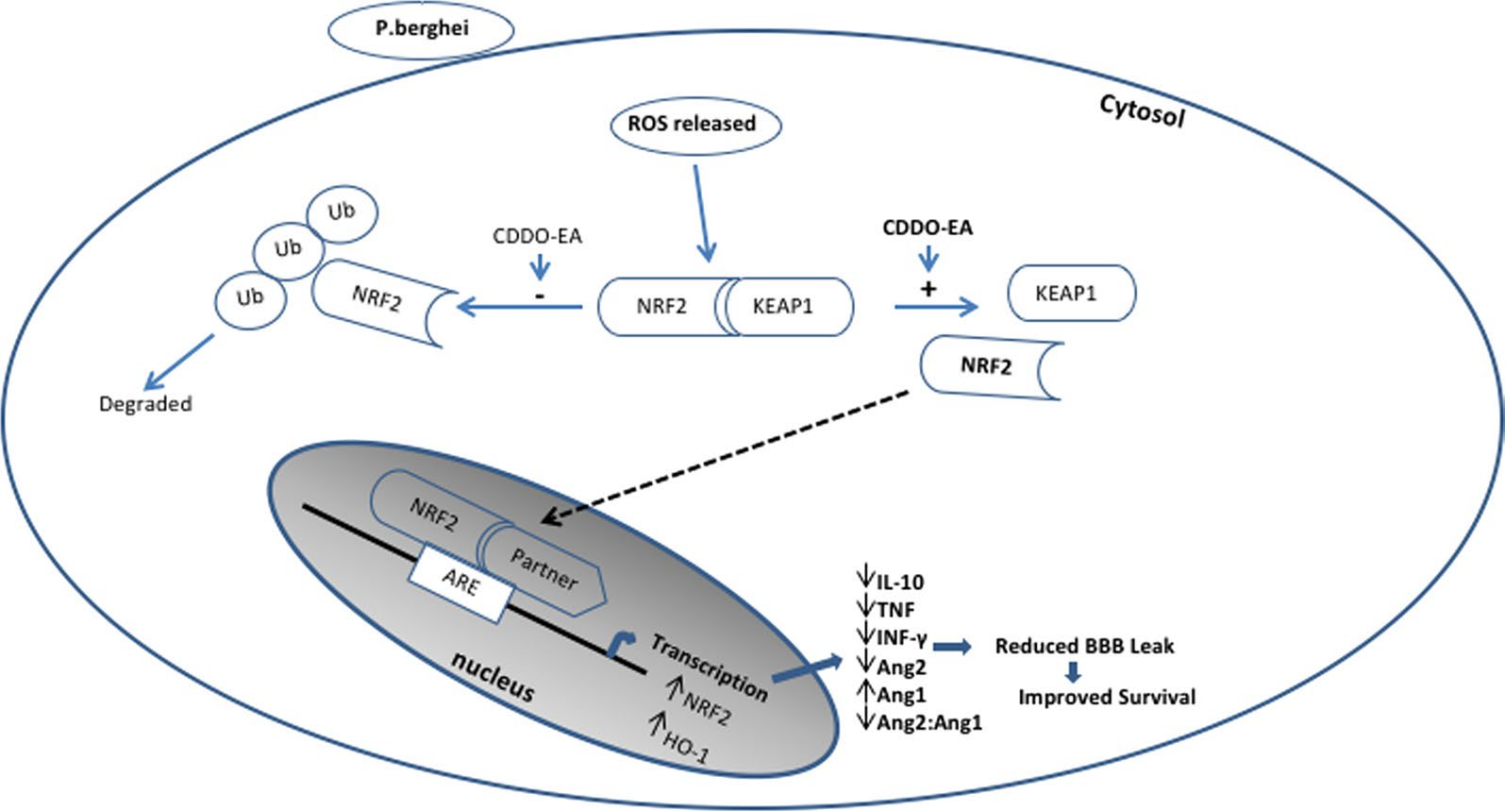
Summary of the protective effects of CDDO-EA in ECM. Infection by *P. berghei* leads to oxidative stress and inflammation. CDDO-EA disrupts the interaction of Nrf2 and Keap1, allowing for the translocation of Nrf2 to the nucleus. With its binding partners, Nrf2 initiates the transcription of genes containing ARE elements in their promoters. In ECM, CDDO-EA treatment reduced levels of IL-10, TNF, INF-γ, Ang-2 while increasing levels of Ang-1, resulting in reduced BBB leak and improved survival

The angiopoietin-Tie2 axis is a critical regulator of endothelial integrity and microvascular leak [69]. Ang-1 binds to and induces the phosphorylation of the Tie2 receptor kinase on endothelial cells. This binding contributes to vascular quiescence and stability. In response to inflammation, Ang-2 is released by endothelial cells, and competitively binds to Tie2, where it promotes vascular permeability and instability [43, 44]. Absolute levels and the ratio between Ang-1 and Ang-2 are associated with disease severity and fatality in CM [39–41, 70]. Ang-1 has recently been shown to play a mechanistic role in the pathogenesis of cerebral malaria and adjunctive treatment with Ang-1 can improve BBB integrity and survival in experimental CM even in the face of high circulating levels of inflammatory cytokines [37]. Adjunctive treatment with CDDO-EA significantly increased levels of Ang-1, and decreased both the levels Ang-2 and the ratio of Ang-2/Ang-1 compared to vehicle control alone. These findings are consistent with the hypothesis that CDDO-EA may improve survival of PbA-infected mice at least in part by via Ang-1 enhanced endothelial and BBB integrity (Fig. 7).

Reduced BBB leak was observed in mice treated with CDDO-EA. Intravital microscopy in PbA-infected mice has shown that neurological symptoms of ECM are associated with breakdown of the neuro-immunological BBB [45] and disruption of microvascular permeability in humans has been associated with fatal CM [8, 42, 71]. CDDO-EA was initially synthesized to increase BBB permeability and has been shown to cross the BBB in mice [33]. Mice with established infection that were treated with CDDO-EA before the onset of neurological symptoms showed improved survival and did not develop neurological signs of ECM. Adjunctive treatment with CDDO-EA before the onset of neurological symptoms significantly reduced BBB leak above that of artesunate treatment alone and prevented mice from developing neurological signs of ECM, as assessed by the RMBCS.

To mimic a more clinically relevant scenario where patients often seek treatment late in the course of disease, mice were treated after the onset of neurological symptoms with adjunctive CDDO-EA co-administered with artesunate. We did not assess CDDO-EA as a monotherapy at this stage as parenteral artesunate is the current first line treatment for severe malaria [72, 73]. This treatment increased survival and improved the neurological outcome of mice already displaying neurological signs, suggesting that adjunctive CDDO-EA may not only prevent BBB leak but also restore its function and mitigate associated neurological injury. Rapid reversibility of cerebral swelling has also been observed in adults and children after anti-malarial treatment and highlights the need for new malarial treatments that that can rapidly prevent and reverse neuronal cytotoxicity [71]. These findings are in agreement with neuroprotective effects observed with triterpenoids used in pre-clinical models of Huntington’s disease and induced neurotoxicity [74, 75].

CDDO-EA did not reduce peripheral parasitaemia levels and all mice that survived ECM went on to develop hyperparasitaemia and anaemia. This indicates that CDDO-EA does not have an anti-parasitic effect on PbA in vivo and its mechanism of action is via modifying host response to infection. These observations are consistent with a growing literature in both pre-clinical models and human infections that host response is a major determinant of outcome and that interventions targeting host response may improve survival and reduce disease severity over antimicrobial therapy alone [76, 37, 40].

CDDO is a partial agonist of PPARγ and this activity may also contribute to improved survival in ECM. CDDO acts by preventing the binding of a nuclear receptor co-repressor (NCoR) and by recruiting CCAAT/enhancer-binding protein (CBP/p300), a transcriptional co-activator to PPARγ [29]. Rosiglitazone, a PPARγ agonist, has been investigated as an adjunctive therapy in ECM where it improves survival, reduces inflammation, prevents vascular leak, and prevents memory and learning deficits in mice with ECM [49]. A randomized, double blind, placebo-controlled trial of rosiglitazone in young adults with uncomplicated malaria resulted in increased parasite clearance times and increased circulating levels of endothelial stabilizing and neuroprotective factors, including Ang-1 and brain-derived neurotrophic factor (BDNF), respectively [51]. A safety and tolerability of rosiglitazone in children with uncomplicated malaria has been completed [72] and is currently being extended to a Phase IIb clinical trial of paediatric severe malaria in Mozambique.

CDDO and its derivatives are currently being investigated in murine models of neurodegenerative disorders such as Amyotrophic lateral sclerosis (ALS) and Huntington’s disease where improvements to memory and motor impairments have been observed [33, 75]. Whether the improved survival in ECM observed with adjunctive CDDO-EA will also be accompanied by reduced cognitive and motor impairments in surviving mice is unknown. It will be important to explore the potential neuroprotective effects of CDDO-EA in future studies. In particular, since CDDO derivatives have been reported to be neuroprotective in several preclinical models, the unique attribute of CDDO-EA to overcome the artemisinin inhibition of Nrf2 may be of utility as an adjunctive therapy for CM.

## Conclusion

In summary, single dose treatment with CDDO-EA improved the survival of ECM-susceptible mice when administered before the development of ECM. This improved survival was associated with increased cerebral expression of Nrf2, reduced levels of TNF and IFN-γ, improved vascular integrity and reduced BBB leak. Importantly CDDO-EA improved survival and disease progression when administered after the onset of neurological symptoms. These results support investigation of CDDO-EA and other SO as potential adjunctive therapy for severe malaria.

## Abbreviations

ALS: amyotrophic lateral sclerosis
Ang: angiopoietin
ARE: antioxidant responsive element
ART: artesunate
BBB: blood brain barrier
CDDO: 2-cyano-3,12-dioxooleana-1,9-dien-28-oic acid
CM: cerebral malaria
DHA: dihydroartesmisinin
EA: ethyl amide
ECM: experimental cerebral malaria
ELISA: enzyme-linked immunosorbent assay
GAPDH: glyceraldehyde 3-phosphate dehydrogenase
HO-1: haem oxygenase-1
IFN-γ: interferon-gamma
IL-10: interleukin-10
Nrf2: nuclear factor (erythroid-derived 2)-like 2
PbA: *Plasmodium berghei* ANKA
PBMC: peripheral blood mononuclear cell
PPARγ: peroxisome proliferator-activated receptor gamma
RMCBS: rapid murine coma and behaviour score
SO: synthetic oleanane triterpenoids
TNF: tumour necrosis factor

## References

[1] WHO. World malaria report 2016. Geneva: World Health Organization; 2016.

[2] Roucher C, Rogier C, Dieye-Ba F, Sokhna C, Tall A, Trape JF (2012). Changing malaria epidemiology and diagnostic criteria for *Plasmodium falciparum* clinical malaria. PLoS ONE.

[3] Griffin JT, Ferguson NM, Ghani AC (2014). Estimates of the changing age-burden of *Plasmodium falciparum* malaria disease in sub-Saharan Africa. Nat Commun..

[4] Fowkes FJI, Boeuf P, Beeson JG (2016). Immunity to malaria in an era of declining malaria transmission. Parasitology.

[5] Fernando SD, Rodrigo C, Rajapakse S (2010). The, “hidden” burden of malaria: cognitive impairment following infection. Malar J..

[6] Brown H, Rogerson S, Taylor T, Tembo M, Mwenechanya J, Molyneux M (2001). Blood–brain barrier function in cerebral malaria in Malawian children. Am J Trop Med Hyg.

[7] Dorovini-Zis K, Schmidt K, Huynh H, Fu W, Whitten RO, Milner D (2011). The neuropathology of fatal cerebral malaria in Malawian children. Am J Pathol.

[8] Cunnington AJ, Walther M, Riley EM (2013). Piecing together the puzzle of severe malaria. Sci Transl Med..

[9] Lamb TJ, Brown DE, Potocnik AJ, Langhorne J (2006). Insights into the immunopathogenesis of malaria using mouse models. Expert Rev Mol Med.

[10] Engwerda C, Belnoue E, Gruner AC, Renia L (2005). Experimental models of cerebral malaria. Curr Top Microbiol Immunol.

[11] de Souza JB, Hafalla JCR, Riley EM, Couper KN (2010). Cerebral malaria: why experimental murine models are required to understand the pathogenesis of disease. Parasitology.

[12] Lou J, Lucas R, Grau GE (2001). Pathogenesis of cerebral malaria: recent experimental data and possible applications for humans. Clin Microbiol Rev.

[13] Hunt NH, Grau GE, Engwerda C, Barnum SR, van der Heyde H, Hansen DS (2010). Murine cerebral malaria: the whole story. Trends Parasitol..

[14] Strangward P, Haley MJ, Shaw TN, Schwartz JM, Greig R, Mironov A (2017). A quantitative brain map of experimental cerebral malaria pathology. PLoS Pathog.

[15] Birbeck GL, Molyneux ME, Kaplan PW, Seydel KB, Chimalizeni YF, Kawaza K (2010). Blantyre Malaria Project Epilepsy Study (BMPES) of neurological outcomes in retinopathy-positive paediatric cerebral malaria survivors: a prospective cohort study. Lancet Neurol..

[16] John CC, Bangirana P, Byarugaba J, Opoka RO, Idro R, Jurek AM (2008). Cerebral malaria in children is associated with long-term cognitive impairment. Pediatrics.

[17] Boivin MJ, Bangirana P, Byarugaba J, Opoka RO, Idro R, Jurek AM (2007). Cognitive impairment after cerebral malaria in children: a prospective study. Pediatrics.

[18] Shabani E, Ouma BJ, Idro R, Bangirana P, Opoka RO, Park GS (2017). Elevated cerebrospinal fluid tumor necrosis factor is associated with acute and long-term neurocognitive impairment in cerebral malaria. Parasite Immunol.

[19] Ssenkusu JM, Hodges JS, Opoka RO, Idro R, Shapiro E, John CC (2016). Long-term behavioral problems in children with severe malaria. Pediatrics.

[20] Woodrow CJ, White NJ (2017). The clinical impact of artemisinin resistance in Southeast Asia and the potential for future spread. FEMS Microbiol Rev.

[21] Hill RA, Connolly JD (2012). Triterpenoids. Nat Prod Rep.

[22] Huang MT, Ho CT, Wang ZY, Ferraro T, Lou YR, Stauber K (1994). Inhibition of skin tumorigenesis by rosemary and its constituents carnosol and ursolic acid. Cancer Res.

[23] Nishino H, Nishino A, Takayasu J, Hasegawa T, Iwashima A, Hirabayashi K (1988). Inhibition of the tumor-promoting action of 12-*O*-tetradecanoylphorbol-13-acetate by some oleanane-type triterpenoid compounds. Cancer Res.

[24] Liby KT, Sporn MB (2012). Synthetic oleanane triterpenoids: multifunctional drugs with a broad range of applications for prevention and treatment of chronic disease. Pharmacol Rev.

[25] Liby KT, Yore MM, Sporn MB (2007). Triterpenoids and rexinoids as multifunctional agents for the prevention and treatment of cancer. Nat Rev Cancer.

[26] Kansanen E, Kuosmanen SM, Leinonen H, Levonenn AL (2013). The Keap1-Nrf2 pathway: mechanisms of activation and dysregulation in cancer. Redox Biol..

[27] Liby K, Hock T, Yore MM, Suh N, Place AE, Risingsong R (2005). The synthetic triterpenoids, CDDO and CDDO-imidazolide, are potent inducers of heme oxygenase-1 and Nrf2/ARE signaling. Cancer Res.

[28] Kobayashi M, Yamamoto M (2006). Nrf2-Keap1 regulation of cellular defense mechanisms against electrophiles and reactive oxygen species. Adv Enzyme Regul.

[29] Wang Y, Porter WW, Suh N, Honda T, Gribble GW, Leesnitzer LM (2000). A synthetic triterpenoid, 2-cyano-3,12-dioxooleana-1,9-dien-28-oic acid (CDDO), is a ligand for the peroxisome proliferator-activated receptor gamma. Mol Endocrinol.

[30] Nagaraj S, Youn J-I, Weber H, Iclozan C, Lu L, Cotter MJ (2010). Anti-inflammatory triterpenoid blocks immune suppressive function of MDSCs and improves immune response in cancer. Clin Cancer Res.

[31] Hong DS, Kurzrock R, Supko JG, He X, Naing A, Wheler J (2012). A phase I first-in-human trial of bardoxolone methyl in patients with advanced solid tumors and lymphomas. Clin Cancer Res.

[32] Sporn MB, Liby KT, Yore MM, Fu L, Lopchuk JM, Gribble GW (2011). New synthetic triterpenoids: potent agents for prevention and treatment of tissue injury caused by inflammatory and oxidative stress. J Nat Prod.

[33] Neymotin A, Calingasan NY, Wille E, Naseri N, Petri S, Damiano M (2011). Neuroprotective effect of Nrf2/ARE activators, CDDO ethylamide and CDDO trifluoroethylamide, in a mouse model of amyotrophic lateral sclerosis. Free Radic Biol Med..

[34] Percário S, Moreira D, Gomes B, Ferreira M, Gonçalves A, Laurindo P (2012). Oxidative stress in malaria. Int J Mol Sci.

[35] Serghides L, Patel SN, Ayi K, Lu Z, Gowda DC, Liles WC (2009). Rosiglitazone modulates the innate immune response to *Plasmodium falciparum* infection and improves outcome in experimental cerebral malaria. J Infect Dis.

[36] Carroll RW, Wainwright MS, Kim KY, Kidambi T, Gómez ND, Taylor T (2010). A rapid murine coma and behavior scale for quantitative assessment of murine cerebral malaria. PLoS ONE.

[37] Higgins SJ, Purcell LA, Silver KL, Tran V, Crowley V, Hawkes M (2016). Dysregulation of angiopoietin-1 plays a mechanistic role in the pathogenesis of cerebral malaria. Sci Transl Med..

[38] Pamplona A, Ferreira A, Balla J, Jeney V, Balla G, Epiphanio S (2007). Heme oxygenase-1 and carbon monoxide suppress the pathogenesis of experimental cerebral malaria. Nat Med.

[39] Lovegrove FE, Tangpukdee N, Opoka RO, Lafferty EI, Rajwans N, Hawkes M (2009). Serum angiopoietin-1 and -2 levels discriminate cerebral malaria from uncomplicated malaria and predict clinical outcome in African children. PLoS ONE.

[40] Erdman LK, Dhabangi A, Musoke C, Conroy AL, Hawkes M, Higgins S (2011). Combinations of host biomarkers predict mortality among Ugandan children with severe malaria: a retrospective case-control study. PLoS ONE.

[41] Conroy AL, Hawkes M, McDonald CR, Kim H, Higgins SJ, Barker KR (2016). Host biomarkers are associated with response to therapy and long-term mortality in pediatric severe malaria. Open Forum Infect Dis..

[42] Seydel KB, Kampondeni SD, Valim C, Potchen MJ, Milner DA, Muwalo FW (2015). Brain swelling and death in children with cerebral malaria. N Engl J Med.

[43] Miller LH, Ackerman HC, Su X, Wellems TE (2013). Malaria biology and disease pathogenesis: insights for new treatments. Nat Med.

[44] Obermeier B, Daneman R, Ransohoff RM (2013). Development, maintenance and disruption of the blood-brain barrier. Nat Med.

[45] Nacer A, Movila A, Baer K, Mikolajczak SA, Kappe SHI, Frevert U (2012). Neuroimmunological blood brain barrier opening in experimental cerebral malaria. PLoS Pathog.

[46] Liby K, Royce DB, Williams CR, Risingsong R, Yore MM, Honda T (2007). The synthetic triterpenoids CDDO-methyl ester and CDDO-ethyl amide prevent lung cancer induced by vinyl carbamate in A/J mice. Cancer Res.

[47] Yates MS, Tauchi M, Katsuoka F, Flanders KC, Liby KT, Honda T (2007). Pharmacodynamic characterization of chemopreventive triterpenoids as exceptionally potent inducers of Nrf2-regulated genes. Mol Cancer Ther.

[48] Aleksunes LM, Goedken MJ, Rockwell CE, Thomale J, Manautou JE, Klaassen CD (2010). Transcriptional regulation of renal cytoprotective genes by Nrf2 and its potential use as a therapeutic target to mitigate cisplatin-induced nephrotoxicity. J Pharmacol Exp Ther.

[49] Kwak M-K, Itoh K, Yamamoto M, Kensler TW (2002). Enhanced expression of the transcription factor Nrf2 by cancer chemopreventive agents: role of antioxidant response element-like sequences in the nrf2 promoter. Mol Cell Biol.

[50] Hawkes MT, Conroy AL, Opoka RO, Hermann L, Thorpe KE, McDonald C (2015). Inhaled nitric oxide as adjunctive therapy for severe malaria: a randomized controlled trial. Malar J..

[51] Serghides L, McDonald CR, Lu Z, Friedel M, Cui C, Ho KT (2014). PPARγ agonists improve survival and neurocognitive outcomes in experimental cerebral malaria and induce neuroprotective pathways in human malaria. PLoS Pathog.

[52] Kossodo S, Monso C, Juillard P, Velu T, Goldman M, Grau GE (1997). Interleukin-10 modulates susceptibility in experimental cerebral malaria. Immunology.

[53] Niikura M, Inoue SI, Kobayashi F (2011). Role of interleukin-10 in malaria: focusing on coinfection with lethal and nonlethal murine malaria parasites. J Biomed Biotechnol..

[54] Eckwalanga M, Marussig M, Tavares MD, Bouanga JC, Hulier E, Pavlovitch JH (1994). Murine AIDS protects mice against experimental cerebral malaria: down-regulation by interleukin 10 a T-helper type 1 CD4 + cell-mediated pathology. Proc Natl Acad Sci USA.

[55] Getachew Y, Cusimano FA, Gopal P, Reisman SA, Shay JW (2016). The synthetic triterpenoid RTA 405 (CDDO-EA) halts progression of liver fibrosis and reduces hepatocellular carcinoma size resulting in increased survival in an experimental model of chronic liver injury. Toxicol Sci.

[56] Kurtzhals JAL, Adabayeri V, Goka BQ, Akanmori BD, Oliver-Commey JO, Nkrumah FK (1998). Low plasma concentrations of interleukin-10 in severe malarial anaemia compared with cerebral and uncomplicated malaria. Lancet.

[57] Wilson NO, Bythwood T, Solomon W, Jolly P, Yatich N, Jiang Y (2010). Elevated levels of IL-10 and G-CSF associated with asymptomatic malaria in pregnant women. Infect Dis Obstet Gynecol..

[58] Zhang G, Manaca MN, McNamara-Smith M, Mayor A, Nhabomba A, Berthoud TK (2012). Interleukin-10 (IL-10) polymorphisms are associated with IL-10: production and clinical malaria in young children. Infect Immun.

[59] Hugosson E, Montgomery SM, Premji Z, Troye-Blomberg M, Björkman A (2004). Higher IL-10 levels are associated with less effective clearance of *Plasmodium falciparum* parasites. Parasite Immunol.

[60] Mandala WL, Msefula CL, Gondwe EN, Drayson MT, Molyneux ME, MacLennan CA (2017). Cytokine profiles in Malawian children presenting with uncomplicated malaria, severe malarial anemia, and cerebral malaria. Clin Vaccine Immunol.

[61] Schofield L, Grau GE (2005). Immunological processes in malaria pathogenesis. Nat Rev Immunol.

[62] Ho M, Sexton MM, Tongtawe P, Looareesuwan S, Suntharasamai P, Webster HK (1995). Interleukin-10 inhibits tumor necrosis factor production but not antigen-specific lymphoproliferation in acute Plasmodium falciparum malaria. J Infect Dis.

[63] King T, Lamb T (2015). Interferon-γ: the Jekyll and Hyde of malaria. PLoS Pathog.

[64] Augustin HG, Young Koh G, Thurston G, Alitalo K (2009). Control of vascular morphogenesis and homeostasis through the angiopoietin–Tie system. Nat Rev Mol Cell Biol.

[65] Jain V, Lucchi NW, Wilson NO, Blackstock AJ, Nagpal AC, Joel PK (2011). Plasma levels of angiopoietin-1 and -2 predict cerebral malaria outcome in Central India. Malar J..

[66] Mohanty S, Benjamin LA, Majhi M, Panda P, Kampondeni S, Sahu PK (2017). Magnetic resonance imaging of cerebral malaria patients reveals distinct pathogenetic processes in different parts of the brain. mSphere..

[67] Dondorp A, Nosten F, Stepniewaska K, Day N (2005). White N and South East Asian Quinine Artesunate Malaria Trial (SEAQUAMET) group. Artesunate versus quinine for treatment of severe falciparum malaria: a randomised trial. Lancet.

[68] Dondorp AM, Fanello CI, Hendriksen IC, Gomes E, Seni A, Chhaganal KD (2010). Artesunate versus quinine in the treatment of severe falciparum malaria in African children (AQUAMAT): an open-label, randomised trial. Lancet.

[69] Stack C, Ho D, Wille E, Calingasan NY, Williams C, Liby K (2010). Triterpenoids CDDO-ethyl amide and CDDO-trifluoroethyl amide improve the behavioral phenotype and brain pathology in a transgenic mouse model of Huntington’s disease. Free Radic Biol Med..

[70] Yang L, Calingasan NY, Thomas B, Charturvedi RK, Kiaei M, Wille EJ (2009). Neuroprotective effects of the triterpenoid, CDDO methyl amide, a potent inducer of Nrf2-mediated transcription. PLoS ONE.

[71] Boggild AK, Krudsood S, Patel SN, Serghides L, Tangpukdee N, Katz K (2009). Use of peroxisome proliferator-activated receptor gamma agonists as adjunctive treatment for *Plasmodium falciparum* malaria: a randomized, double-blind, placebo-controlled trial. Clin Infect Dis.

[72] Varo R, Crowley VM, Sitoe A, Madrid L, Serghides L, Bila R (2017). Safety and tolerability of adjunctive rosiglitazone treatment for children with uncomplicated malaria. Malar J..

[73] Dumont M, Wille E, Calingasan NY, Tampellini D, Williams C, Gouras GK (2009). Triterpenoid CDDO-methylamide improves memory and decreases amyloid plaques in a transgenic mouse model of Alzheimer’s disease. J Neurochem.

[74] Elphinstone R, Riley F, Lin T, Higgins S, Dhabangi A, Musoke C (2015). Dysregulation of the haem-haemopexin axis is associated with severe malaria in a case–control study of Ugandan children. Malar J..

[75] Elphinstone RE, Besla R, Shikatani EA, Lu Z, Hauslden A, Davies M (2017). S-nitrosoglutathione reductase (GSNOR) deficiency confers improved survival and neurological outcome in experimental cerebral malaria. Infect Immun.

[76] Gazzinelli RT, Kalantari P, Fitzgerald KA, Golenbock DT (2014). Innate sensing of malaria parasites. Nat Rev Immunol.

